# Splenic Infarction in Acute Cytomegalovirus and Human Parvovirus Concomitant Infection

**DOI:** 10.1155/2018/7027656

**Published:** 2018-12-03

**Authors:** Tomoya Harada, Yuriko Sueda, Kensaku Okada, Tsuyoshi Kitaura, Kosuke Yamaguchi, Haruhiko Makino, Masaki Nakamoto, Hiroki Chikumi, Akira Yamasaki

**Affiliations:** ^1^Division of Medical Oncology and Molecular Respirology, Faculty of Medicine, Tottori University, 36-1 Nishi-cho, Yonago, Tottori, Japan; ^2^Division of Infectious Disease, Tottori University Hospital, 36-1 Nishi-cho, Yonago, Tottori, Japan

## Abstract

We present a case report of a 35-year-old woman who had splenic infarction. She had persistent high fever, systemic joint pain, and abnormal liver function. She was diagnosed with cytomegalovirus and human parvovirus B19 concomitant infection. Her coagulopathy test revealed no abnormal results. She was treated with intravenous ganciclovir for 13 days; consequently, her splenic infarction improved after 7 weeks. As per our knowledge, this is the first case of cytomegalovirus and parvovirus B19 coinfection complicated by splenic infarction. Cytomegalovirus and parvovirus B19 may induce a hypercoagulation state during the acute phase.

## 1. Introduction

Infectious mononucleosis is commonly caused by Epstein–Barr virus (EBV) infection, which is characterized by pharyngitis, lymphadenopathy, atypical lymphocytosis, and hepatosplenomegaly. Although cytomegalovirus (CMV) infection is commonly asymptomatic, approximately 10% of adults who are initially infected with CMV present with infectious mononucleosis. CMV is the second most common cause of infectious mononucleosis [1]. Recently, the rate of young adults who are infected with CMV has decreased; in fact, the serological prevalence rate of CMV among pregnant women is approximately 69% in Japan [2]. Human parvovirus B19 is well known for causing erythema infectiosum in children. Nontypical symptoms of polyarthropathy, erythema, and red cell aplasia have been observed in adult infections, and in rare cases, it is the cause of infectious mononucleosis. Although infectious mononucleosis is usually a self-limiting disease and has a good prognosis, several complications, including hematological complications, can occur. Splenic rupture is one of the most serious complications of infectious mononucleosis and occurs in only 0.1% of patients [3]. Splenic infarction rarely occurs in infectious mononucleosis. As per our knowledge, there has been no previous report of splenic infarction due to CMV and parvovirus coinfection.

Herein, we report the case of a young woman with infectious mononucleosis-like syndrome who developed splenic infarction during the acute phase of CMV and parvovirus B19 concomitant infection. After treatment with ganciclovir, her splenic infarction improved.

## 2. Case Presentation

**Figure 1:**
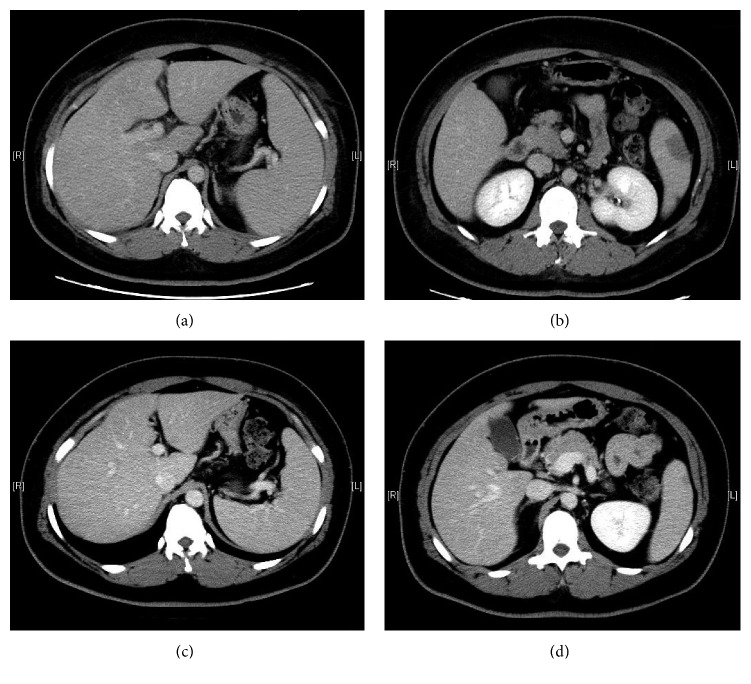
Abdominal computed tomography at admission. (a) Enlarged liver and spleen are shown. (b) A geographical low attenuation area is observed on the dorsal side of the spleen, which suggests splenic infarction. These findings were improved after ganciclovir treatment. (c) Enlarged liver and splenomegaly were improved. (d) The geographic low attenuation area in the spleen was decreased.

A 35-year-old woman was referred to our hospital after having high fever and systemic joint pain for 10 days. She was admitted to our hospital for further examination and treatment. She had a past medical history of diabetes mellitus, herpes zoster, and lichen planus; her family history was unremarkable. On admission, her body temperature was 38.3°C, her blood pressure was 119/90 mmHg, her pulse rate was 110 beats/min, and the oxygen saturation in the room air was 97%. Erythematous papule appeared on the anterior chest and bilateral forearm. She had no pharyngitis, lymphadenopathy, or abdominal pain. Her total white blood cell count was 9,200/*µ*L (neutrophils 87%, lymphocytes 7%, and atypical lymphocytes 2%), CD4^+^ T cell was 44.3%, CD8^+^ T cell was 34.5%, hemoglobin was 15.3 g/dL, and platelet count was 123,000/*µ*L. Her laboratory test results were as follows (Table 1): C-reactive protein level, 6.83 mg/dL; aspartate transaminase, 96 U/L; alanine transaminase, 130 U/L; alkaline phosphatase, 489 U/L; *γ*-glutamyl transpeptidase, 349 U/L; lactate dehydrogenase, 315 U/L; total bilirubin, 1.2 mg/dL; HbA1c, 8.7%; immunoglobulin G, 1574 mg/dL; immunoglobulin A, 186 mg/dL; immunoglobulin M, 144 mg/dL. The results of the serological tests for hepatitis B and C were negative. The result of renal function test was normal. Urinalysis showed negative occult blood and urinary protein. The blood cultures showed no growth. Because the patient continued to have high fever, we performed an abdominal computed tomography (CT) scan, which revealed splenomegaly and a geographic low attenuation area in the dorsal and upper external side of the spleen (Figures 1(a) and 1(b)); this finding was consistent with splenic infarction.

We considered the splenic infarction to be in a hypercoagulable state. Her coagulopathy tests showed the following results: prothrombin time, 14.9 sec (normal, 10.6–14.9 sec); activated partial thromboplastin time, 33.9 sec (normal, 23.3–38.2 sec); d-dimer, 6.4 *µ*g/mL; lupus anticoagulant, 1.05 (normal values <1.2, with dilute Russell's viper venom method); anticardiolipin antibody IgG, <8.0 U/mL; and anticardiolipin-*β*2-glycoprotein I complex antibody, <1.2 U/mL. There were no abnormal coagulopathy results.

**Figure 2:**
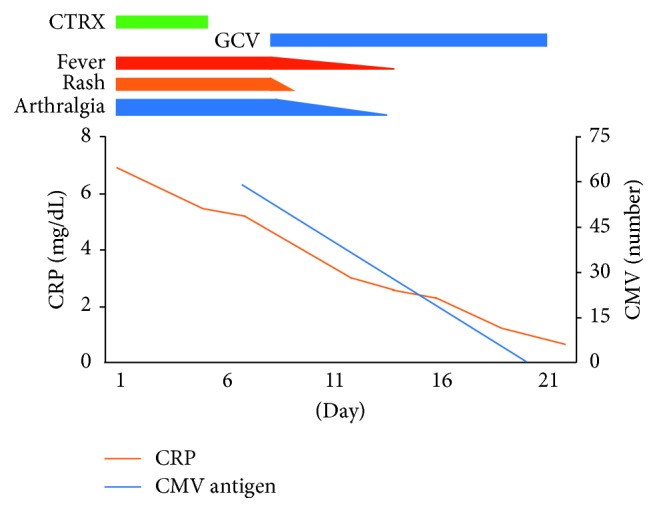
Clinical course of this case. CTRX: ceftriaxone; GCV: ganciclovir; CRP: C-reactive protein; CMV: cytomegalovirus.

Because of atypical lymphocytosis and elevated transaminase, infectious mononucleosis was considered the cause of her sustained fever. The serological test for EBV antiviral capsid antigen IgM was negative at 0.1 (normal level, <0.5), antiviral capsid antigen IgG was positive at 7.5 (normal level, <0.5), and antibody to EBV nuclear antigen was positive at 4.6 (normal level, <0.5). These results suggest past infection for EBV. The serological test for CMV IgM was positive at 4.60 (normal level, <0.8), CMV IgG was slightly positive at 2.5 (normal level, <2.0), and parvovirus B19 IgM was also slightly positive at 0.99 (normal level, <0.8). The CMV antigen pp65 (antigenemia method of C10C11) was detectable at a level of 33 positive cells per 150,000 cells and 25 positive cells per 150,000 cells, which confirmed her diagnosis of acute CMV infection. The results of the human immunodeficiency virus type 1 (HIV-1) antigen/antibody and human T-cell leukemia virus type-1 (HTLV-1) antibody were negative. Although the patient was originally treated with conservative therapy, her fever persisted; therefore, she was subsequently treated with intravenous ganciclovir. The day after beginning the treatment with ganciclovir, her fever, joint pain, and erythematous papule improved (Figure 2). After 13 days of ganciclovir therapy, the CMV antigen pp65 was decreased at a level of 1 positive cells per 150,000 cells and she was discharged. Two weeks after the first serological tests of the virus antibodies, a follow-up serological test was performed. CMV IgM was positive at 4.80, CMV IgG was elevated to 11.7, and parvovirus B19 IgM was elevated to 2.38. Acute parvovirus infection was confirmed by the increasing level of parvovirus B19 IgM antibody. Seven weeks after discharge, we performed an additional abdominal CT scan. The splenic infarction area was decreased without atrophy, and splenomegaly was improved (Figures 1(c) and 1(d)).

## 3. Discussion

Splenic infarction is an uncommon disorder that occurs when splenic artery branches become occluded, either by an embolus or thrombus. The causes of splenic infarction are cardiogenic emboli such as atrial fibrillation, autoimmune diseases such as antiphospholipid syndrome, hematological diseases, and infection. Various infectious diseases, such as varicella zoster virus, HIV, and malaria, were previously reported to cause splenic infarction. The hypercoagulable state caused by these diseases can induce thromboembolic complications.

Infectious mononucleosis is mainly caused by EBV. Other viruses such as CMV, human herpes virus type 6, parvovirus B19, and HIV-1 can be associated with infectious mononucleosis-like syndrome with atypical lymphocytes [1]. The most widely used diagnostic criterion of infectious mononucleosis is Hoagland's criterion. For patients presenting with clinically suspected infectious mononucleosis and at least a 50% lymphocytosis and 10% atypical lymphocytes, the diagnosis should be confirmed by the positive serologic test to EBV. But these criteria are not high sensitivity [4]. Although present case had high fever, splenomegaly, detected viral antigen, and increased in titer of antibodies to CMV and parvovirus B19, atypical lymphocytes were only 2%. We diagnosed this case as infectious mononucleosis-like syndrome caused by CMV and parvovirus B19. The incidence of splenic infarction as a complication of infectious mononucleosis is extremely rare. A 10-year retrospective case series revealed that 49 patients developed splenic infarction out of approximately 60,000 total patients [5]. Only 2 of those 49 patients were diagnosed with infectious mononucleosis caused by EBV. Another retrospective case series showed only 32 patients with splenic infarction, corresponding to a frequency of 0.016% over 10 years of admissions [6]. Although 4 patients were diagnosed with infection-associated splenic infarction in that study, no patients were diagnosed with infectious mononucleosis.

In previously reported cases of infectious mononucleosis with splenic infarction, the most abnormal coagulopathy result was the transient appearance of antiphospholipid antibodies during the acute phase of infectious mononucleosis as a result of EBV [7–9]. In addition, CMV infection was reported to induce thromboembolisms, such as portal vein thrombosis and splenic infarction [10–12]. The most reported mechanism was also the transient appearance of antiphospholipid antibodies [13]. Uthman and Gharavi showed that anticardiolipin antibodies and lupus anticoagulant were associated with viral infections such as hepatitis C virus, EBV, CMV, and parvovirus B19 [14]. Thromboses associated with CMV were primarily reported in immunocompromised patients, such as acquired immunodeficiency syndrome patients and transplant recipients. Human parvovirus B19 infection induced several unusual clinical manifestations, such as vasculitis, myocarditis, glomerulonephritis, immune thrombocytopenia, and hemophagocytic syndrome. Splenic infarction is an extremely rare complication of parvovirus B19 infection. To our knowledge, four cases of thromboembolism associated with parvovirus B19 infection have been previously described [15–18]. Although these occurrences resulted in transient antiphospholipid antibodies, one case did not have elevated titers of antiphospholipid antibodies, and the mechanism of thromboembolism was unknown in that case [15].

In nearly all cases of splenic infarction during viral infection, transient antiphospholipid antibodies have been documented. Although this may be the pathophysiological mechanism of splenic infarction, antiphospholipid antibodies were not detected in the present case. Another theory suggests that CMV infects endothelial cells and enhances the expression of adhesion molecules and tissue factors on their surfaces. This action results in platelet adhesion and aggregation on vessel walls. Activation of factor *X*, increasing levels of von Willebrand factor, and factor VIII are other theories of the mechanism behind CMV-induced thrombosis [19]. Viral infection in itself may change the anticoagulant environment of the vascular endothelium to favor coagulation. Khoretonenko et al. showed that mice infected with CMV displayed an impaired arteriolar vasodilator response and that CMV induced transient leukocyte and platelet adhesion in postcapillary venules [20]. These results indicated that CMV infection induced microvascular dysfunction. Because the present case was infected with CMV and parvovirus B19 simultaneously, it was supposed that the environment of the vascular endothelium displayed a greater change in coagulation.

Antopolsky et al. showed that diabetes mellitus was presented in 23% of the patients who developed splenic infarction; therefore, diabetes mellitus is considered one of the risk factors for thrombosis [5]. Although this case presented poorly controlled diabetes mellitus, splenic infarction was improved by treatment with ganciclovir. It was indicated that viral infections were the main cause of splenic infarction in this case. However, poorly controlled diabetes mellitus might affect the clinical course of present case. Cytomegalovirus infection is often a self-limiting disease; however, this case had continued high fever for about 3 weeks and even needed treatment of ganciclovir. Although we did not measure severity biomarker such as cytokines and galectin, the severe clinical course indicated that hypercytokinemia might be developed and might affect coagulopathy.

The route of infection in this case was equivocal, because she does not have any children of her own. But she lived in complex housing where many families lived with children and she had frequent contact with these children. Furthermore, she suffered from poor-controlled diabetes mellitus that induced immunocompromised state. She might infect CMV and parvovirus B19 in this community, and poor controlled diabetes mellitus might affect infection structure.

In conclusion, this is the first documented case of CMV and human parvovirus B19 concomitant infection complicated with splenic infarction in which antiphospholipid antibodies were not detected. Physicians should be aware of thromboembolisms during the acute phase of viral infections, particularly in patients infected with more than one virus.

## Figures and Tables

**Table 1:** Clinical data of this case.

Hematology		Serology		Biochemistry	
White blood cell	9200/*μ*L	CRP	6.83 mg/dL	Total bilirubin	1.2 mg/dL
Neutrophil	87%	IgG	1574 mg/dL	AST	96 U/L
Lymphocyte	7%	IgA	186 mg/dL	ALT	130 U/L
Atypical lymph	2%	IgM	144 mg/dL	ALP	489 U/L
Hemoglobin	15.3 g/dL	C3	122.0 mg/dL	γ-GTP	349 U/L
Platelet	123,000/*μ*L	C4	22.1 mg/dL	LDH	315 U/L
CD4	44.3%	CH50	66.5 U/mL	BUN	8.4 mg/dL
CD8	34.5%	Ferritin	459 ng/mL	Cr	0.47 mg/dL
CD3	77.9%	sIL-2R	1129 U/mL	HbA1c	8.7%
CD19	9.6%	EBV-VCA IgM	0.1	HBs-Ag	<0.001 IU/mL
		EBV-VCA IgG	7.5	HCV-Ab	0.1 COI
		EBV-EBNA IgG	4.6	HTLV-1 Ab	0.1 COI
		CMV IgM	4.6	HIV-1 Ag/Ab	0.2 COI
		CMV IgG	2.5		
		Parvovirus B19-IgM	0.99		

CRP: C-reactive protein, IgG: immunoglobulin G, IgA: immunoglobulin A, IgM: immunoglobulin M, sIL-2R: soluble IL-2 receptor, EBV: Epstein–Barr virus, VCA: virus capsid antigen, EBNA: EBV nuclear antigen, CMV: cytomegalovirus, AST: aspartate transaminase, ALT: alanine transaminase, ALP: alkaline phosphatase, *γ*-GTP: *γ*-glutamyl transpeptidase, LDH: lactate dehydrogenase, BUN: blood urea nitrogen, Cr: creatinine, HBs-Ag: hepatitis B surface antigen, HCV-Ab: hepatitis C virus antibody, HTLV-1 Ab: human T-cell leukemia virus type-1 antibody, HIV-1 Ag/Ab: human immunodeficiency virus type-1 antigen/antibody.

## References

[1] Naito T., Kudo N., Inui A. (2006). Causes of infectious mononucleosis-like syndrome in adult patients. *Internal Medicine*.

[2] Shigemi D., Yamaguchi S., Otsuka T. (2015). Seroprevalence of cytomegalovirus IgG antibodies among pregnant women in Japan from 2009–2014. *American Journal of Infection Control*.

[3] Bartlett A., Williams R., Hilton M. (2016). Splenic rupture in infectious mononucleosis: a systematic review of published case reports. *Injury*.

[4] Ebell M. H. (2004). Epstein-Barr virus infectious mononucleosis. *American Family Physician*.

[5] Antopolsky M., Hiller N., Salameh S., Goldshtein B., Stalnikowicz R. (2009). Splenic infarction: 10 years of experience. *American Journal of Emergency Medicine*.

[6] Schattner A., Adi M., Kitroser E., Klepfish A. (2015). Acute splenic infarction at an academic general hospital over 10 Years: presentation, etiology, and outcome. *Medicine (Baltimore)*.

[7] Cull E., Stein B. L. (2012). Splenic infarction, warm autoimmune hemolytic anemia and antiphospholipid antibodies in a patient with infectious mononucleosis. *International Journal of Hematology*.

[8] Van Hal S., Senanayake S., Hardiman R. (2005). Splenic infarction due to transient antiphospholipid antibodies induced by acute Epstein-Barr virus infection. *Journal of Clinical Virology*.

[9] Naviglio S., Abate M. V., Chinello M., Ventura A. (2016). Splenic infarction in acute infectious mononucleosis. *Journal of Emergency Medicine*.

[10] Rawla P., Vellipuram A. R., Bandaru S. S., Raj J. P. (2017). Splenic infarct and pulmonary embolism as a rare manifestation of cytomegalovirus infection. *Case Reports in Hematology*.

[11] Wang T., Kuttikat A., Pulsalkar P., Nanguzgambo A., Bhalara S. (2015). Cytomegalovirus-associated portal vein thrombosis in an immunocompetent patient: an underestimated complication. *Oxford Medical Case Reports*.

[12] Ladd A. M., Goyal R., Rosainz L., Baiocco P., DiFabrizio L. (2009). Pulmonary embolism and portal vein thrombosis in an immunocompetent adolescent with acute cytomegalovirus hepatitis. *Journal of Thrombosis and Thrombolysis*.

[13] Poon M. L., Tang J. W., Chee Y. L. (2012). Case report: cytomegalovirus-induced thrombosis in an immunocompetent patient. *Journal of Medical Virology*.

[14] Uthman I. W., Gharavi A. E. (2002). Viral infections and antiphospholipid antibodies. *Seminars in Arthritis and Rheumatism*.

[15] Kranidiotis G., Efstratiadis E., Kapsalakis G., Loizos G., Bilis A., Melidonis A. (2016). Splenic infarcts as a rare manifestation of parvovirus B19 infection. *IDCases*.

[16] Reitblat T., Drogenikov T., Sigalov I., Oren S., London D. (2000). Transient anticardiolipin antibody syndrome in a patient with parvovirus B19 infection. *American Journal of Medicine*.

[17] Asano Y., Sarukawa M., Idezuki T. (2006). Multiple small pulmonary emboli associated with transient antiphospholipid syndrome in human Parvovirus B19 infection. *Clinical Rheumatology*.

[18] Tanizawa K., Nakatsuka D., Tanaka E. (2009). Pulmonary thrombosis with transient antiphospholipid syndrome after mononucleosis-like illness. *Internal Medicine*.

[19] Sherman S., Eytan O., Justo D. (2014). Thrombosis associated with acute cytomegalovirus infection: a narrative review. *Archives of Medical Science*.

[20] Khoretonenko M. V., Leskov I. L., Jennings S. R., Yurochko A. D., Stokes K. Y. (2010). Cytomegalovirus infection leads to microvascular dysfunction and exacerbates hypercholesterolemia-induced responses. *American Journal of Pathology*.

